# Clinical significance of circulating tumor cells in gastric cancer patients

**DOI:** 10.18632/oncotarget.14879

**Published:** 2017-01-28

**Authors:** Jitao Zhou, Xin Ma, Feng Bi, Ming Liu

**Affiliations:** ^1^ Department of Medical Oncology/Laboratory of Signal Transduction & Molecular Targeted Therapy, West China Hospital, Sichuan University, Chengdu, Sichuan, China

**Keywords:** CTC, gastric cancer, EMT, Cellsearch, cancer metastasis

## Abstract

Circulating tumor cells (CTCs) are rare cancer cells released from tumors into the blood stream that are thought to have a key role in cancer metastasis. Investigation of CTCs is an exciting area of research but remains in its infancy, and the presence of CTCs has been associated with worse prognosis in several major cancer types. Gastric cancer (GC) is a highly lethal malignancy and a serious public health concern in East Asia especially in China. There is an urgent need for identifying new, better prognostic markers to enhance diagnosis and prognosis, facilitate drug development, and to improve the treatment of gastric cancer patients. There are considerable interests in gastric CTCs given their potential use as gastric cancer biomarkers. This review highlights recent advances in studies of gastric CTCs, including the isolation and biological molecular characteristics of gastric CTCs, and their clinical significance.

## INTRODUCTION

Gastric cancer (GC) is a highly lethal malignancy and a serious public health concern in East Asia, Eastern Europe, Central and South America. It was the fourth most common cancer (950000 new cases per year) and the third leading cause of cancer death all around the world in 2012 (GLOBOCAN 2012) [1]. Despite recent improvement in diagnosis and therapeutic methods, the 5-year survival rate is still less than 30% [2]. Approximately 50% of GC patients suffer from tumor recurrence or metastasis after curative resection [3]. For the patients diagnosed with advanced gastric cancer (AGC), chemotherapy and target therapy are mainstays of treatment. Unfortunately, more than 40% patients with AGC have no response to chemotherapy and the others would acquire chemo-resistance soon, which leads to both poor survival and limited therapeutic options [4]. Therefore, there is an urgent need for identifying new, better prognostic markers to facilitate the diagnosis, improve the prediction of relapse and metastasis, and to predict the prognosis and determine the therapeutic responsiveness in AGC patients.

Disseminated tumor cells in the peripheral blood of cancer patients are known as circulating tumor cells (CTCs). CTCs were first identified by Thomas Ashworth in 1869 [5]. However, the CTC research has been hampered by the technical difficulties to detect these rare cells. It was not until decades later that numerous new approaches were established to isolate, enumerate, and characterize CTCs in patients with various cancers [6]. CTCs have attracted more and more attention not only because of their easy accessibility but also their superiority over common tumor markers. CTCs represent both the phenotypic and genetic compositions of primary and metastatic tumors. Recent studies have shown that CTCs can be found in many solid tumor types such as lung, breast, renal, bladder, head and neck, and colorectal cancers [7–16]. In some of the solid cancers, enumeration of CTCs has served as surrogate markers for overall survival (OS), risk of relapse, progression-free survival (PFS) and chemotherapy effectiveness [7–17]. The most recent efforts have focused on haematological spread, epithelial-mesenchymal transition (EMT), cellular cooperation, circulating tumour microemboli (CTM) and the tumor-initiating capability of CTCs [18–25].

The aims of this review are to provide a comprehensive overview on the studies of CTCs in GC, including the biology, detection methods, and the clinical significance of CTCs in GC patients, discuss challenges facing the field, and to elaborate future prospects.

## DETECTION OF CTCS IN PATIENTS WITH GC

CTCs have been detected in the blood of cancer patients at a very low concentration of 1 cell per 5×10^9^ red cells and 5-10×10^6^ white blood cells [26]. Therefore, extremely sensitive and specific methods are required for detecting and enumerating CTCs generated by various malignancies including GC [17]. Generally, CTCs detection is a two-step procedure: first enrichment and then detection [27]. The enrichment step includes morphologic-based isolation and immune-magnetic selection (e.g. magnetic affinity cell sorting or magnetic beads enrichment) [28]. The enrichment step captures CTCs without labelling, using properties such as density (gradient centrifugation), cell size (membrane filters), electric charge (dielectrophoresis) and deform ability (microfluidic system in a chip) [29–32]. The detection step includes using either nucleic acid-based assays (reverse transcription polymerase chain reaction, RT-PCR) or cell recognition-based assays.

In the early days, only RT-PCR was used to identify and detect CTCs [33–34]. Identification of appropriate markers expressed by CTCs is critical to enhance the specificity and reliability of its detection. Conventional markers such as CKs and CEA mRNA were most used to detect CTCs in GC patients [35–37]. However, these tumor markers are expressed in normal peripheral blood cells and epithelial cells. Thus, some new biomarkers expressed in CTCs such as survivin and B7-H3 have been found to better predict tumor progression or prognosis in gastric cancer patients [38–40]. Multiple markers have been used to avoid false positive detection and increase detection rate. The detection of c-Met or MUC1 mRNA may be a promising tool for early detection of micro-metastatic CTCs in GC patients [41]. The combination of four markers (human telomerase reverse transcriptase (TERT), cytokeratin 19 (CK19), CEA and MUC1) serves as a prognostic indicator for postoperative recurrence/metastasis and overall survival in GC [42]. In general speaking, RT-PCR is highly sensitive, reproducible and economically feasible. But the specificity of this method is hindered by incalculability of CTCs and high false positive rates due to illegitimate marker expression by normal cells [43–44].

Cell-recognition based assays represent a major advancement in detecting CTCs. After the enrichment step, CTCs are detected using immunocytometric assays, such as immunocytochemistry, immunofluorescence and flow cytometry [45]. Comparing to RT-PCR, cell-recognition based assays allow enumeration of CTCs, morphological analysis of stained cells and molecular characterization of extracted cells [46]. Recently, the CellSearch system has been approved by U.S.FDA for CTC enumeration to improve prognosis and treatment efficacy in breast, colorectal and prostate cancer [47–49]. The platform is a semi-automated methodology able to detect and enumerate CTCs. 7.5 ml of whole blood is mixed with magnetic iron nanoparticles coated with anti-EpCAM antibody. Then EpCAM positive cells are stained with anti-CK and anti-CD45 fluorescent antibodies to detect epithelial cells and to exclude leukocytes, respectively. DAPI nuclear dye (4′,6-diamidino-2-phenylindole) is used to allow microscopic identification of relevant cell fraction. CTCs are identified as CK^+^DAPI^+^CD45^−^. By using this method, a number of studies have reported the role of CTCs in GC patients.

Alternative approaches are in development to improve detection rate by isolating CTCs with high purity. One such approach is called CTC-chip consisting of an array of antibody (EpCAM)-coated microposts and has successfully identified CTCs in the peripheral blood of patients with metastatic lung, prostate, pancreatic, breast and colon cancer samples [50]. Another approach is called “Herringbone-chip” created by the same research team using herringbone grooves within the transparent wall of the device allowing for higher blood volume throughput and increases CTC capture efficiency and purity [51].

## BIOLOGY OF CTCS IN PATIENTS WITH GC

Recently emerging evidences suggest that there is considerable heterogeneity in CTC populations. This feature of CTCs has potential of informing intra-tumor heterogeneity and tumor evolution and thus has attracted more and more attention of research community [27, 52].

### Circulating gastric cancer stem cells

A subset of cancer cells are thought to have the properties of adult stem cells (self-renewal and multipotency) and are capable of initiating tumor growth. These cells have been named as ‘cancer stem cells’ or ‘tumor-initiating cells’. CTCs with stem cell-like characteristics might play a crucial role in breast cancer metastasis [53–54]. CD44 has been identified as a marker of gastric cancer stem cells [55]. In a prospective study of 45 GC patients, nineteen CTC-positive patients had CD44-positive CTCs, and they were more likely to develop metastasis and recurrence than patients with CD44-negative CTCs. Moreover, the mean time to recurrence was shorter in patients with CD44-positive CTCs. Thus, CD44 positivity in CTCs might serve as a novel marker for predicting metastasis and recurrence risk for GC patients [56–57]. Consistent with this notion, our group has found that compared to CD44^−^/CD45^−^ gastric CTCs, CD44^+^/CD45^−^ gastric CTCs have stronger colony formation ability and exhibit higher capacity of migration, invasion and resistance to irradiation, chemotherapy drugs and target drugs [58].

### GC CTCs and EMT

Most cancer-related deaths are associated with metastasis. CTCs are known to be necessary but not sufficient for the initiation of metastasis. EMT is a multistep process that plays a key role in metastasis and cancer progression [59]. EMT is characterized by the downregulation of epithelial markers, such as EpCAM and CK, and the upregulation of mesenchymal markers, such as vimentin and twist. CTCs that have undergone EMT can escape the detection by the EpCAM-based enrichment technique like CellSearch system [60]. Although several studies explored mesenchymal markers (vimentin, N-cadherin, O-cadherin, twist, fibronectin and serpin peptidase inhibitor) to profile the EMT phenotype of CTCs, no defined marker or panel of markers have been identified [61].

Li et al [62] detected CTCs of 44 GC patients for the expression of four epithelial (E+) transcripts (keratins 8, 18, and 19 and epithelial cell adhesion molecule) and two mesenchymal (M+) transcripts (Vimentin and Twist) by a quantifiable, dual-colorimetric RNA-*in situ* hybridization assay. CTCs had been captured in 35 GC patients and EMT CTCs were identified in 14 patients with M+ or M+ > E+. Our group also found that epithelial markers pan-CK, E-cadherin were decreased, and mesenchymal markers N-cadherin, vimentin were overexpressed in gastric CTCs [63]. This EMT subgroup of CTCs in gastric cancer deserves more attention in the future.

### Molecular characterization of GC CTCs and target therapy

Ideally, CTCs are used as a liquid biopsy to identify the molecular features of a patient's tumor. Then accordingly, the results may guide personalized treatment options. Some small sample studies have been carried out to verify this hypothesis [64–66]. For example, HER2 expression in CTCs of breast cancer has been evaluated. Interestingly, some breast cancer patients have discordant HER2 status for their CTCs (positive) and primary tumor (negative), but they still benefit from trastuzumab therapy [67–68].

Karyotyping and phenotyping of the enriched CTCs in therapeutic advanced GC patients were carried out by using the integrated subtraction enrichment (SET) and immunofluorescence staining *in situ* hybridization (iFISH) platform. CellSearch system was applied as a reference control. Phenotyping of CTCs in HER2 positive AGC patients demonstrated that HER2+ CTCs could be effectively eliminated in response to HER2-targeted therapy. Karyotyping of CTCs indicated that examination of the copy number of chromosome 8 in CTCs provides a potential approach for predicting chemotherapeutic efficacy and monitoring chemo-resistance [69]. However, the relevance of CTC heterogeneity in response to or in developing resistance to targeted therapy is yet to be unveiled. More well-designed and prospective randomized studies assessing the predictive value of CTCs are needed.

## CLINICAL UTILITY OF CTCS IN PATIENTS WITH GC

The detection of CTCs in peripheral blood has received growing enthusiasm in the clinical utility of various cancers. Since 1990s many studies have been performed to indicate the correlation between CTCs and the clinicopathological characteristics of gastric tumor [33–42, 69–71]. These studies suggest that CTCs may play a role in predicting tumor prognosis and response to treatment. However, these studies used a variety of methods for CTC detection. Tang et al [72] have performed a meta-analysis of the diagnostic accuracy of various CTCs detection methods in gastric cancer and found that results vary among different detection methods. They believe that a solid conclusion could be drawn with the development of new detection technologies.

To date, the CellSearch system is the only CTCs detecting technique approved by the U.S.FDA. It is possible to obtain highly reproducible quantitative results from different laboratories by using this detection platform. CTC numbers as quantified by the CellSearch procedure have been shown to have prognostic significance, predicting chemotherapy effectiveness in patients with various types of carcinomas (e.g. breast cancer, prostate cancer and colorectal cancer) [7–11]. The current review will focus on studies reporting significant data about the role of CTCs in gastric cancer obtained by the CellSearch system (Table 1).

**Table 1:** Summary of relevant results from CTC studies in gastric cancer by CellSearch

Characteristic and number of patientsy	Detection rate	Positive cutoff	Results	Ref.
Nonmetastatic 14 Metastatic 27 Post curative resection 3	14.3% 55.6% 0	≥2CTCs	Correlated with advanced tumor stage, peritoneal dissemination, shorter survival. The change in CTCs correlate with disease progression and chemotherapeutic effect.	Hiraiwa K [69]
Metastatic 52 baseline 2 week 4 week	nonPD vs PD 19.2%vs 13.4% 0 vs 13.7% 2% vs 16.7%	≥4CTCs	CTCs measurement may be useful as a surrogate marker for determining response to S-1 based or paclitaxel regimens in advanced GC.	Matsusaka S [75]
Resection 148 Nonresectable 103	60.2% 11.3%	≥1CTCs	Predicting tumor progression, prognosis, and the effect of chemotherapy in patients with gastric cancer.	Uenosono Y [73]
Advanced GC 136	18.4%	≥1CTCs	Independent predictor of a shorter PFS in AGC. A valuable biomarker for selecting patients required intensive treatment.	Okabe H [74]
Advanced GC 136 106 evaluable Patients baseline 6 week	41.9% 42.5% 24.5%	≥3CTCs	Post-therapy CTC level evaluating therapeutic response and predicting their prognosis. Changes in CTCs following therapy useful in rapidly identifying ineffective treatments and poor prognosis.	Yilin Li [76]

GC: gastric cancer; PD: progression disease; CTCs: circulating tumor cells

Measurement of CTCs in GC patients could be useful for judging tumor stage and predicting patients’ survival. Yoshikazu et al [73] performed a prospective study to evaluate CTCs in 251 GC patients and explored the clinical impact of CTCs using the CellSearch system. CTCs were detected in 16 patients (10.8%) from the resection group with a significantly higher relapse rate, in 62 patients (60.2%) from the nonresectable group but not detected in healthy volunteers. Furthermore, CTCs in patients who underwent gastrectomy were significantly correlated with the depth of tumor invasion, lymph node metastasis, distant metastasis, disease stage, vessel invasion, and lymphatic invasion. Consistent with the above, the overall survival rate of the entire cohort with CTCs was significantly lower than that of patients without CTCs. Hiraiwa et al [69] performed a similar study enrolled with 130 gastrointestinal cancer patients and 41 healthy volunteers, including 44 gastric cancer patients. CTC counts were larger in 27 metastatic GC patients than that in 14 non-metastatic patients. Another study including 136 patients with AGC suggests that emergence of CTCs is an independent predictor of a shorter PFS [74]. Thus, evaluation of CTCs may be a useful strategy for predicting tumor progression and prognosis in patients with GC.

Besides being as diagnostic and prognostic indicators, CTCs hold promise to be biomarkers to aid in treatment selection. The detection of CTCs in the peripheral blood of patients with AGC has been reported to predict efficacy of chemotherapy treatment. In another study, CTCs of whole blood at baseline, 2 weeks, and 4 weeks were detected in 52 patients with AGC treated with S-1-based regimen (S-1 with or without cisplatin) or paclitaxel. Patients with ≥4 CTCs at 2-week and 4-week points had much shorter PFS and OS than those with < 4 CTCs [75.] A single-centre, prospective study was undertaken in 136 AGC patients and their CTCs were enumerated at baseline and at the first response evaluation. Following 6 weeks of chemotherapy, the elevated CTCs may correspond with an ineffective therapeutic response and independently predict a shorter PFS and OS [76]. Monitoring dynamic changes of CTCs in response to therapy may be a useful alternative for assessing treatment resistance.

Measurement of CTCs may be a useful diagnostic tool to predict pleural dissemination which is difficult to detect in current imaging studies, such as computed tomography, ultrasonography and positron emission tomography. Breast cancer, prostate cancer or colorectal cancer patients who have hematogenous metastasis seem to have a high incidence of CTCs. However, by the CellSearch system, CTCs were detected more often in GC patients with peritoneal metastasis(PM)compared to GC patients with hematogenous metastasis [74]. The underlying mechanism remains unclear. Considering that detection of CTCs by the CellSearch system is based on isolation and enumeration of cells expressing EpCAM, EpCAM appears to play an important role in peritoneal metastasis (PM). Imano et al [77] have found that all of 35 PM specimens showed overexpression of EpCAM. Thus they postulate that only GC cells expressing a high level of EpCAM might metastasize to the peritoneum. Meanwhile, the EpCAM antibody has been reported to be a therapeutic agent in patients with malignant ascites in several types of carcinoma [78].

## PERSPECTIVES

In summary, the science of CTCs is an exciting area of research but remains in its infancy. The translation of CTC research to a routine clinical test is hurdled by the lack of consensus in technical approaches. The CellSearch system is the first standard semi-automated methodology approved by U.S.FDA to detect and enumerate CTCs. A number of studies have reported that enumeration of CTCs using the CellSearch system could serve as an indicator of prognosis and treatment efficacy in various cancers, such as breast, colorectal and prostate cancers. Recently, several studies reported that measurement of CTCs with the CellSearch in GC patients could be utilized for judging tumor stage, predicting patients’ survival and evaluating therapeutic response. However, large-scale clinical trials would be desirable for further validating the significant role of CTCs in GC patients and for determining the proper cutoff of positive CTCs score for GC patients.

CTCs are now defined to be a group of heterogeneous cells and have been suggested to have great potential for personalized therapy selection. This will be the key direction in CTCs research field. More studies are still needed to understand the CTC biology and determine the biomarkers of different subgroup of CTCs (e.g. EMT transition cells, stem cells). The CellSearch technology has limitation in detecting EMT CTCs. Novel revolutionary approaches must have increased sensitivity to accommodate CTC heterogeneity and need to be assessed in clinical trials to gain a position in clinical practice.

## Abbreviations

CTC: Circulating Tumor Cells
GC: Gastric Cancer
AGC: Advanced Gastric Cancer
EMT: Epithelial-Mesenchymal Transition
CTM: Circulating Tumour Microemboli
OS: Overall Survival
PFS: Progression-Free Survival
HER2: Human Epidermal Growth Factor Receptor 2
